# Factors Influencing Insulin Absorption Around Exercise in Type 1 Diabetes

**DOI:** 10.3389/fendo.2020.573275

**Published:** 2020-10-21

**Authors:** Jason P. Pitt, Olivia M. McCarthy, Thomas Hoeg-Jensen, Benjamin M. Wellman, Richard M. Bracken

**Affiliations:** ^1^Applied Sport, Technology, Exercise and Medicine Research Centre (A-STEM), College of Engineering, Swansea University, Swansea, United Kingdom; ^2^Diabetes Peptide and Protein Chemistry, Novo Nordisk A/S, Maaloev, Denmark

**Keywords:** absorption, insulin, exercise, pharmacokinetics, type 1 diabetes (T1D), subcutaneous tissue, physiology

## Abstract

International charities and health care organizations advocate regular physical activity for health benefit in people with type 1 diabetes. Clinical expert and international diabetes organizations’ position statements support the management of good glycemia during acute physical exercise by adjusting exogenous insulin and/or carbohydrate intake. Yet research has detailed, and patients frequently report, variable blood glucose responses following both the same physical exercise session and insulin to carbohydrate alteration. One important source of this variability is insulin delivery to the circulation. With modern insulin analogs, it is important to understand how different insulins, their delivery methods, and inherent physiological factors, influence the reproducibility of insulin absorption from the injection site into circulation. Furthermore, contrary to the adaptive pancreatic response to exercise in the person without diabetes, the physiological and metabolic shifts with exercise may increase circulating insulin concentrations that may contribute to exercise-related hyperinsulinemia and consequent hypoglycemia. Thus, a furthered understanding of factors underpinning insulin delivery may offer more confidence for healthcare professionals and patients when looking to improve management of glycemia around exercise.

## Introduction

**Figure 1:**
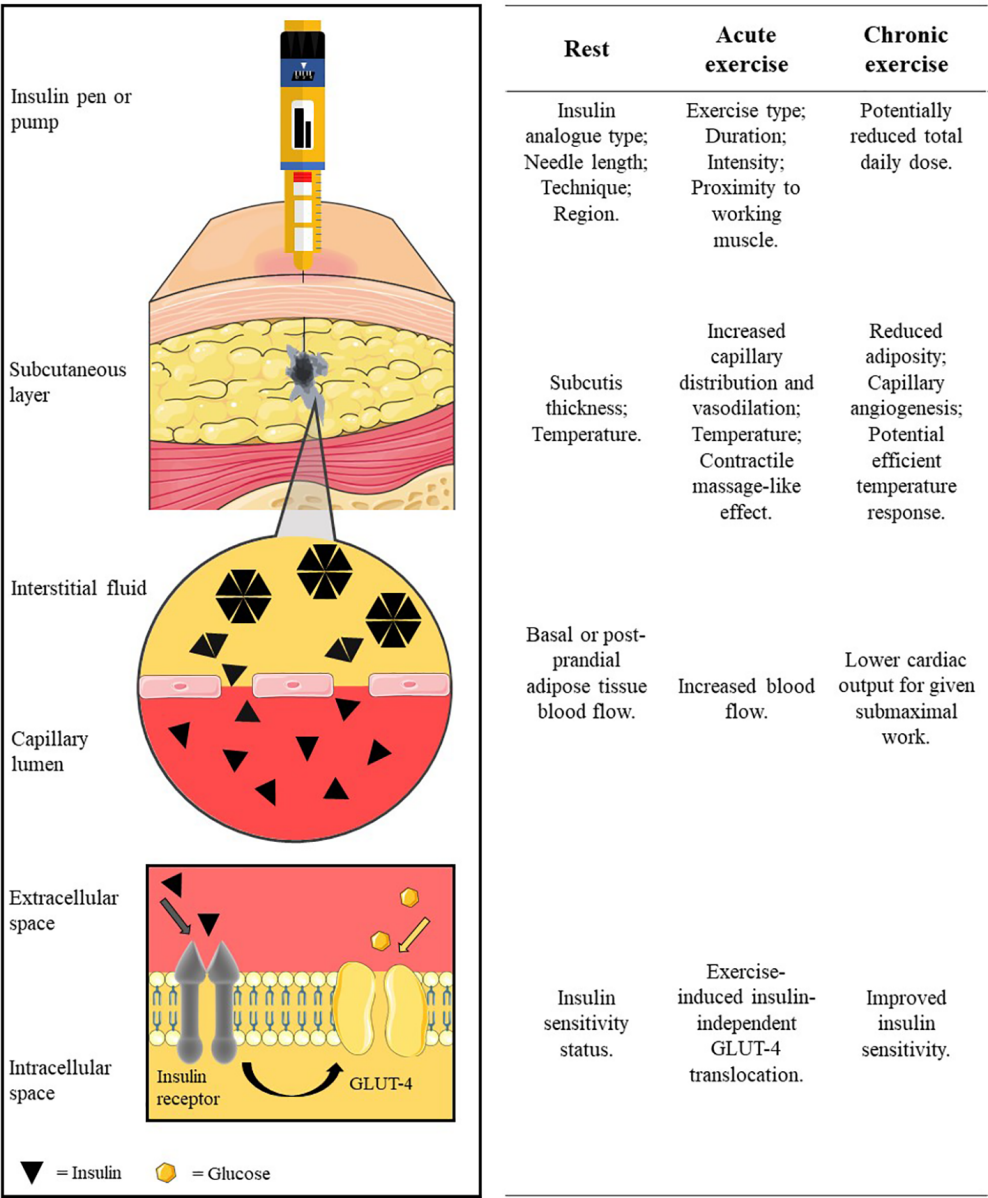
The pathway of subcutaneously administered exogenous insulin. Insulin is injected/released from formulation in the insulin pen/pump cartridge into the subcutaneous tissue. The insulin oligomers disassociate into monomer units before translocating across the capillary endothelium into blood circulation. Insulin circulates before binding to an insulin receptor to facilitate glucose uptake into the cell (e.g. into the myocyte). Factors at rest, acute exercise, and chronic exercise which affect each stage are listed along the row beside each illustrated stage of the pathway. Insulin diffusion in the subcutaneous layer is adapted with permission from digital images of insulin depot formation 15 to 30 s after bolus injection into porcine subcutaneous tissue; authored by Jockel et al. (1). Image is not to scale for illustration purposes. Created using Servier Medical Art (https://smart.servier.com/); Sevier Medical Art by Servier is licensed under a Creative Commons Attribution 3.0 Unported License.

People with type 1 diabetes (T1D) are required to administer exogenous insulin *via* multiple daily insulin (MDI) regimen or automated pump therapy. For both delivery methods, insulin is administered into adipose tissue that lies beneath the dermal layers of the skin and above the musculoskeletal compartment. Upon entering this subcutaneous layer, the injected insulin forms a depot, where it is at its highest concentration in the interstitium. Insulin spreads in the subcutaneous layer, following the path of least resistance around adipocytes and along loose connective tissue, towards the capillary system (1) (**Figure 1**). Separating the interstitium and the capillary lumen is the vascular endothelium which is poorly penetrated by hexameric insulin—the typical state in which it is stored. Insulin molecules must dissociate into smaller monomer units to move across the capillary endothelium and enter the circulation. The rate at which insulin molecules can move from the initial depot to being physiologically available in the bloodstream determines the insulin’s profile of metabolic activity.

It is of clinical concern when the *same* insulin regimen exhibits a varied glucose-lowering effect for the patient injecting insulin. Intraindividual variability in an insulin’s absorption compromises the patient’s ability to predict blood glucose concentrations, thereby increasing the risk of fluctuations outside the physiologic range (i.e. ≤3.9 - ≥10.0 mmol.L^−1^) (2). This is especially pertinent to the individual with T1D who is engaging in structured exercise or spontaneous bouts of physical activity. The response to acute exercise induces significant changes in physiological systems which have the potential to alter the environment in which exogenous insulin is administered into, affecting its absorption kinetics and subsequent circulatory appearance. To aid in reproducing good glucose control, and mitigate the risk of exercise-induced hypoglycemia, it is important to be aware of the effects of exercise on the rate of insulin absorption (from injection depot into circulation).

This review aims to (i) inform the reader of relevant factors that influence insulin absorption at rest and (ii) critique the research investigating exercise effects on insulin absorption, providing an evidence-based explanation of the underlying mechanisms, where possible.

## Exogenous Insulin

At concentrations necessary for subcutaneous injection, human insulin self-associates into hexamer crystals which are too large to pass through capillary fenestrae and reach circulation. The significant time required to allow the hexamers in the subcutis to dissociate to monomer units, that are small enough to diffuse into the capillary lumen, results in serum insulin concentrations out of sync with post-absorption blood glucose concentrations. The development of genetic engineering in recombinant DNA technology enabled modifications of insulin’s molecular structure that influence its pharmacokinetic properties. Initial modifications focused on inverting (*Lys^B28^-Pro^B29^*: insulin lispro) (3) and substituting (*Pro^B28^* ➔ *Asp^B28^*: insulin aspart) (4) the amino acid sequence to weaken the van der Waals forces and hydrogen bonds shared at the C-termini B-chains at the dimer interface to decrease self-association, while avoiding alterations of the insulin receptor binding sites of insulin. Development of long-acting insulin has been based on acylation and pH-dependent subcutaneous precipitation (5). Further to structural modifications, excipients can be added to (or removed from) marketed formulations to effect pharmacokinetics. For example, insulin fiasp includes the excipient niacinamide to induce local vasodilation and counter-act the aggregating effect of *Zn^2+^* (6), which promotes faster absorption. Improvements in insulin absorption have also been demonstrated in protease (that prevent enzymatic degradation of insulin) (7) and hyaluronidase (that cleaves hyaluronate polymers at injection site) (8) excipients; however, these are yet to appear in marketed formulations. **Table 1** summarizes different exogenous insulins’ chemical composition and their consequent pharmacokinetic properties.

**Table 1:** Synopsis of the pharmacokinetic properties of exogenous insulin.

Insulin action type	Name	Manufacturer	Chemical modifications	Mechanism	Pharmacokinetic profile			References
					*Duration*	*Onset*	*Peak*	
ULTRA-RAPID	Fiasp (Faster-acting insulin aspart)	NovoNordisk	Niacinamide and L-arginine added to solution (insulin structure is that of insulin aspart)	Niacinamide excipient destabilizes hexamer in subcutis and may mediate local vasodilation	3–5 h	3–5 min	45–60 min	Hövelmann et al. (9)
	Lyumjev (Ultra-rapid lispro)	Eli Lilly and Company	Treprostinil and citrate added to solution (insulin structure is that of lispro)	Citrate increases local vascular permeability and treprostinil increases local vasodilation	5 h	2 min	45–60 min	Leohr et al. (10)
RAPID	Humalog (Lispro)	Eli Lilly and Company	Inverted *Lys* (B29) and *Pro* (B28) sequence	Distortion at the dimer interface destabilizes hexamer	3–5 h	5–20 min	45–60 min	Howey et al. (11)
	Apidra (Glulisine)	Sanofi	*Asn* (B3) replaced with *Lys*, and *Lys* (B29) replaced with *Glu*. Formulation is zinc free.	Lower isoelectric point improves solubility at physiological pH	3–5 h	10 min	45–60 min	Danne et al. (12)
	Novorapid (Aspart)	NovoNordisk	*Pro* (B28) replaced with *Asp* in B-chain	Removing *Pro* (B28) intermolecular contact to *Glu* (B21) and disrupting hydrogen bonds at dimer interface destabilizes hexamer	3–5 h	10 min	45–60 min	Plank et al. (13)
SHORT	Actrapid	NovoNordisk	Regular human insulin	Hexamer formation in storage delays appearance in circulation	8 h	30 min	1–2.5 h	Mortensen et al. (14)
INTERMEDIATE	Novo NPH	NovoNordisk	Protamine added to insulin solution	Formation of crystals in solution	10–14 h	1.5 h	4 h	Lepore et al. (15)
LONG	Levemir (Detemir)	NovoNordisk	C14 fatty acid is bound to *Lys* (B29) and *Thr* (B30) is removed	Human serum albumin binding and dodecamer formation	20–24 h	2.5 h	None	Porcellati et al. (16)
	Lantus (Glargine)	Sanofi	*Asp* (A21) replaced by *Gly*, and adding two *Arg* amino acids onto the B-chain (B31, B32)	Isoelectric point ~7 leads to precipitation in subcutis	20–24 h	1.5 h	None	Lepore et al. (15)
ULTRA-LONG	Degludec (Tresiba)	NovoNordisk	C16 fatty diacid γ-*Glu* is bound to *Lys* (B29) and *Thr* (B30) is removed	Formation of multi-hexamer units	24–42 h	30–90 min	None	Haahr & Heise (17)
	Glargine U300 (Toujeo)	Sanofi	*Asp* (A21) replaced by *Gly*, and adding two *Arg* amino acids onto the B-chain (B31, B32)	Larger precipitate than U100 glargine delays dissolution	>30 h	30–90 min	None	Becker et al. (18)

Arg, Arginine; Asp, aspartic acid; Glu, glutamic acid; Gly, glycine; Lys, lysine; NPH, neutral protamine Hagedorn; Pro, proline; Thr, threonine.

## Physical and Physiological Factors Affecting the Delivery of Insulin

### Subcutaneous Tissue

The subcutaneous tissue consists primarily of adipocytes and an extracellular matrix made up of connective tissue and interstitial fluid, which present barriers of different resistances to insulin in its pathway to the vascular system (28). The impedance of insulin movement upon injection is subject to factors influencing the status of the subcutis, namely, the tissue type (injection depth), adipose tissue layer thickness, and temperature (**Figure 1**).

### Injection Depth

Several studies demonstrate the importance of subcutaneous injection in tempering, and reducing the variation of, the pharmacokinetic/pharmacodynamic profiles of injected insulin compared to intramuscular injection at rest (29–31). Additionally, the extent to which the subcutaneous and muscular sites of injection are affected by exercise are not identical, which may result in differing rates of insulin absorption in the injection sites during exercise. An exercise-induced increase in insulin absorption was observed after intramuscular injection of ^125^I-labeled insulin actrapid (human insulin) into the thigh prior to moderate-intensity cycling (intramuscular injection: rest, 0.46 ± 0.08 vs exercise, 1.17 ± 0.14%.min^−1^; *p* < 0.001), but not after subcutaneous injection (rest, 0.31 ± 0.05 vs exercise, 0.45 ± 0.09%.min^−1^; *p* > 0.05) in people with T1D (32). Consequently, there was a greater exercise-induced fall in blood glucose during intramuscular injection (intramuscular: −4.6 ± 0.4 vs subcutaneous: −2.8 ± 0.7 mmol.L^−1^; *p* < 0.05) (32).

Combined with data collected on the thickness of the subcutaneous tissue in patients (33), recent recommendations advocate a transition to shorter needle lengths (such as 4 and 5 mm) to minimize the variability and altered glucodynamic activity of intramuscular injections (34). Further work is needed to determine whether intramuscular injection into the exercising muscle has a different effect on absorption than injection into a non-working muscle.

**Sub-section conclusion:**

The effects of intramuscular insulin are more rapid and variable than subcutaneous insulin injections at rest and during exercise.

### Subcutaneous Tissue Properties

An inverse relationship exists between subcutaneous thickness and the rate of insulin absorption. In healthy participants, weak-moderate negative correlations exist between subcutaneous fat layer thickness and serum insulin appearance rate (35) and a slower time to peak plasma insulin concentrations in those with a higher body mass index (BMI) (> 23.6 kg.m^−2^) by 31 min (95% CI 13.7–48.5; *p* < 0.05) (36). In two separate studies, Vora and colleagues employed similar methodologies, measuring the rate of absorption of ^125^I-labeled insulin actrapid from different injection sites in healthy participants (37) and those with T1D (38), with otherwise matched characteristics. The results from individuals with T1D suggested a weaker inverse correlation between the rate of absorption of insulin and the degree of adiposity than those without T1D. This prompts the questions ‘what causes this difference?’ and ‘is there a difference between the rate of absorption of exogenous insulin in T1D and healthy individuals?’ The use of a range of rate constants in these studies makes comparison between the two population cohorts difficult and warrants further investigation to answer these questions. Differences in fasted blood flow in abdominal subcutaneous fat tissue may contribute to the impact of tissue thickness in healthy and T1D populations (39), but studies that directly compare the structure and absorption characteristics within adipose tissue are lacking.

**Sub-section conclusion:**

Greater subcutaneous adipose tissue layer thickness is associated with a tempered absorption profile of injected insulin.

### Local and Ambient Temperature

The effect of temperature on exogenous insulin absorption has been investigated in those with and without T1D. One study investigated insulin absorption in individuals with T1D who injected insulin actrapid 60 min before two 25-min bouts of sitting in a sauna at 85°C (40). Compared to a control day (22°C), participants experienced a 110% greater disappearance of ^125^I-labeled actrapid from the site of injection during the whole sauna period, corresponding with a significant blood glucose drop of ≥3 mmol.L^−1^ after the sauna (*p* < 0.05). Later results from a pilot trial (41) and a randomized controlled trial (42) supported these findings, showing that after administering insulin aspart *via* pump the time to peak insulin action was faster using a local skin-warming device (40°C) compared to without its use (77 ± 5 vs 111 ± 7 min, respectively; *p* < 0.001). It is interesting to note here that favorable absorption kinetics have been demonstrated during application of both local and ambient heating, as well as in pump and MDI therapy. Using a long-acting insulin, Bitton and colleagues recently reported a trend of a lower drop in glucose from baseline after 6 h of warming the injection site of insulin glargine U100 (−2.2 ± 0.7 mmol.L^−1^) compared to a control group (−1.0 ± 0.6 mmol.L^−1^), yet the difference between the two trials was statistically non-significant (*p* = 0.11) (43).

**Sub-section conclusion:**

Increased ambient temperature or local warming of the injection site accelerates insulin absorption.

## The Impact of Acute Physical Exercise on Insulin Absorption

Muscular exercise induces rapid changes in the physiological systems of the person with type 1 diabetes to supply working muscles with oxygen and nutrients. The exercise pressor reflex (i.e. a peripheral neural reflex arising from skeletal muscle contraction) prompts cardiovascular changes, namely: an increase in cardiac output, blood pressure, and a shunting of blood away from the viscera towards the working muscles, aided by increased concentrations of adrenaline, noradrenaline, and cortisol (44, 45). After rapid adaptation to the exercise intensity, sympatho-adrenal activity is well-regulated relative to the power output during sustained aerobic activities. To maintain normoglycemia, pancreatic insulin secretion decreases and glucagon secretion increases to better match hepatic glucose release to the raised muscular glucose uptake (46).

For the individual with T1D, the physiological changes induced by exercise pose a problem for maintaining glucose control. The synergistic effect of relative hyperinsulinemia (from the previous exogenous injection) and exercise-induced insulin-independent pathways cause the uptake of glucose into myocytes to exceed hepatic glucose release and a decline in blood glucose during continuous steady-state exercise (47). What may further exacerbate the imbalance between glucose uptake and glucose input to the circulation is an exercise-induced acceleration of insulin absorption from the injection site into the blood.

**Table 2** overviews the randomized controlled trials that have compared the rate of insulin absorption in individuals during acute exercise compared to rest. There is considerable variation in the findings that can be broadly separated into studies investigating short- and intermediate-acting insulins and studies on long-acting insulins. The agreement that basal insulin glargine U100 does not produce a rise in absorption during exercise opposes the findings that bolus insulins, such as actrapid, become significantly elevated (**Table 2**). Interestingly, it was found that neutral protamine Hagedorn (NPH), an intermediate-acting insulin, was greatly elevated during exercise compared to resting conditions (24); a rise even more marked than those exhibited by some short-acting insulins in the literature (19, 21, 23, 48). This may, in part, be explained by the longer needle length of 12.7 mm being used, likely creating an insulin depot that is closer to the capillary-dense muscle tissue (see 2.1 *Needle Length* section). In contrast, Kemmer et al. (20) demonstrated no change in insulin absorption rate during continuous exercise using actrapid, a short-acting insulin. It is initially unclear what caused Kemmer and colleagues’ investigation to evidence no change in insulin mobilization during exercise, but it is noteworthy that the methods employed in the study detailed a large (20 U) volume of injected insulin. Larger insulin volumes may result in altered absorption times due to a smaller surface area: volume ratio and slower diffusion rate (49). The reasons for the overall discrepancy between the effects of exercise on short- and intermediate-acting insulin against long-acting insulins are still unclear and warrant further investigation. However, it should be noted that many of the articles included in this review are >30 years old, during which time new insulin analogs have been developed. This is somewhat reflected by the wide use of ^125^I-labeled insulin disappearance measurements. While there is evidence iodo-radioactively labeling insulin analogs slows absorption kinetics, its use is valid when comparing absorption rates within studies (50).

**Table 2:** Randomized controlled trials investigating the effect of exercise compared to rest on insulin absorption in people with type 1 diabetes or healthy individuals.

Authors and date (arrow indicating exercise-induced change in insulin absorption)	Investigated insulin (units injected)	Site of injection	Insulin absorption measurement	Exercise methodology	Insulin absorption outcome
**Short- and intermediate-acting insulins investigated**					
Ferrannini et al. (19) 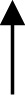	Actrapid (8 U)	Thigh and abdomen	^125^I-labeled actrapid (radioactivity count)	Healthy participants (n = 8; undefined M/F) performed 20 min of moderate-intensity continuous exercise (ending in 170 bpm HR) on cycle ergometer	Increased RIA during exercise in leg injection (exercise 1.12 ± 0.12 vs Rest 0.68 ± 0.15%.min^−1^; p < 0.05). No change in abdomen (exercise 0.87 ± 0.18 vs Rest 0.75 ± 011%.min^−1^; *p*>0.05)
Kemmer et al. (20) 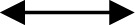	Actrapid (20 U)	Leg and arm (undefined)	^125^I-labeled actrapid (radioactivity count)	Participants with T1D (n=9; M 8/F 1) performed 10 min bouts separated by 5 min rest, for 30 min total exercising, continuous low-to-moderate intensity exercise (125 ± 5 bpm) on cycle ergometer	Increased RIA after exercise in leg injection compared to same time period at rest (undefined, statistically significant); however, no change during exercise. No change in RIA at any timepoint in arm injection compared to rest
Kolendorf et al. (1979) (21) 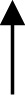	Actrapid (8 U)	Thigh	^131^I-labeled actrapid insulin (radioactivity count)	Participants with T1D (n = 5; undefined M/F) performed four 10-min periods, with 400-sec intervals, of moderate-intensity continuous exercise (120 ± 10 bpm) on cycle ergometer	Increased RIA during exercise compared to rest (Exercise 0.71 ± 0.18 vs Rest 0.41 ± 0.15%.min^−1^; *p* < 0.05)
McAuley et al. (22) 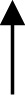	Aspart (pump) (TDD 0.55 ± 0.10 U.kg^−1^.day^−1^)	Abdomen	Venous blood sampling (radioimmunoassay)	Participants with type 1 diabetes (n = 14; M 7/F 7) performed 30 min, including a 5 min warm up, of moderate-intensity continuous exercise (65–70% age-predicted maximal heart rate on a cycle ergometer)	Significant increase of mean free insulin concentration during exercise by 6 ± 2 pmol.L^−1^ compared to rest (*p* < 0.001)
Ronnemaa & Koivisto (23) 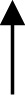	Actrapid (5 ± 1 U)	Thigh	Venous blood sampling (radioimmunoassay)	Participants with type 1 diabetes (C-peptide negative) (n = 9; M 9/F 0) performed three 15-min periods, with 5-min rest intervals, of moderate-intensity continuous exercise (3-min warm-up, then 12-min at 60% VO_2max_) on cycle ergometer, in either cold (10°C) or warm (30°C) ambient temperatures	Significant difference in plasma free insulin (average difference over whole exercise bout) between exercise and rest in 10°C, 2.7 mU.L^−1^ (*p* < 0.01) and 30°C, 3.7 mU.L^−1^ (*p* < 0.05)
Thow et al. (24) 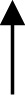	NPH (0.25 U.kg^−1^)	Thigh	Venous blood sampling (radioimmunoassay)	Healthy participants (n=7; M 7/F 0) performed 60 min low-to-moderate-intensity continuous exercise (5 km.h^−1^ at 5° gradient) on treadmill	Increased serum insulin concentration from pre-exercise rest to average peak in exercise (13.7 ± 1.2 vs 27.3 ± 3.2 mU.L^−1^; NSR)
Susstrunk et al. (25) (undefined) 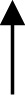	Actrapid (0.12 U.kg^−1^)	Abdomen or Thigh	Venous blood sampling (radioimmunoassay)	Healthy volunteers (n = 4; undefined M/F) performed three 15-min bouts exercise, separated by 5-min rest periods, of continuous exercise at low-to-moderate-intensity (50% maximum power capacity) on a cycle ergometer	Rate of insulin absorption was higher upon injecting into the abdomen (0.039 U.min^−1^) than into the thigh (0.027 U.min^−1^; *p*<0.05). Both sites experienced marginal enhancements of RIA under exercising conditions compared to rest (NDR+NSR)
**Long-acting insulins investigated**					
Peter et al. (3) 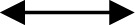	Glargine (27.2 ± 9.1 U)	Thigh	^125^I-labeled Glargine	Participants with type 1 diabetes (n = 13; M 12/F 1) performed 30 min of moderate-intensity continuous exercise (65% VO_2max_) on cycle ergometer	No significant change in RIA between exercise and rest trial days (NDR; *p*=0.548)
Turner et al. (26) 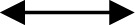	Glargine (27.5 ± 3.1 U)	NDR	Venous blood samples (immunometric assay)	Participants with type 1 diabetes (n = 8; M 7/F 1) performed either control (rest), 1, 2, or 3 sets of moderate to high intensity (67 ± 3% 1RM) resistance exercise	No significant change in plasma insulin concentrations between or within trials (during exercise = NDR, post exercise *p=*0.096)
Turner et al. (27) 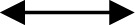	Glargine (27.5 ± 3.1 U)	NDR	Venous blood samples (immunometric assay)	Participants with type 1 diabetes (n = 8; M 7/F 1) performed either control (rest), 1, 2, or 3 sets of moderate-to-high intensity (60–70% 1RM) resistance exercise	No significant change in plasma insulin concentrations between any exercise trials and control, at any timepoints after exercise (during exercise = NDR)

bpm, beats per minute; F, females; HR, heart rate; M, males; NDR, no data reporting; NPH, neutral protamine Hagedorn insulin; NSR, no statistical reporting; RIA, rate of insulin absorption; RM, repetition maximum; TDD, total daily dose; U, units (of insulin); VO_2max_, peak rate of oxygen uptake.

Increasing exercise workload to a high intensity, such as heavy weightlifting in resistance exercise, is associated with large increases in adrenaline and noradrenaline levels, in addition to high rates of H^+^ generation and efflux from muscle cells. In people with T1D, elevations in catecholamine concentrations stimulate hepatic glucose release to a degree which exceeds muscular glucose uptake, contributing to an increase in blood glucose that contrasts the decline typically observed at lower intensities. Turner and colleagues conducted two studies from which point-concentrations of plasma insulin can be compared between resistance exercise protocols and a rested control session (26, 27). It can be assumed from these studies that the total insulin measurements obtained reflected glargine concentrations, as participants were c-peptide negative and omitted their prior bolus insulin dose on the morning of the trial. Both studies found no exercise-induced change in insulin concentrations (**Table 2**). This finding is pertinent to the basal insulin glargine (U100) being measured, as its long-acting profile is dependent on its precipitation at the higher pH (~7.4) of human subcutis compared to marketed formulation (pH ~4.0) (51). Despite a resistance exercise-induced nadir in venous blood pH of ~7.2, the pharmacokinetics of insulin glargine were unaltered compared to control group.

**Sub-section conclusion:**

Physical exercise increases the rate of insulin absorption in intermediate-, short-, and rapid-acting insulins but not in older long-acting insulins. There remains a dearth in the literature studying this effect in modern insulins and with exercise modalities other than sub-maximal endurance activities.

The protracted mechanism of action of intermediate- and long-acting insulins is primarily dependent on the slowed movement and delayed dissociation of insulin oligomers into monomeric form to cross the endothelial layer into systemic circulation. Exercise has limited influence on the rate of insulin dissociation, and consequent availability for absorption, as its initial location is confined to the subcutaneous interstitium (48). Hence, the molecular structure of insulin oligomers is the initial rate-limiting factor in its translocation across the capillary membrane, preceding any influence of exercise. Furthermore, the influence of exercise on insulin absorption is likely negatively correlated with the specific insulin analog duration of action (i.e. a lesser effect on long-acting insulins). For example, a bout of exercise lasting for a guideline-recommended time of 30 min (52), overlaps with, and accelerates the rate of absorption during, a greater segment of insulin action in a rapid-acting insulin (e.g. insulin aspart: time until peak onset of action = 31–70 min, time of duration of action = 3–5 h (53)) compared to a long-acting insulin (e.g. insulin degludec: peakless, time of duration of action > 24 h (54)). The more rapid shift to monomer units in short-acting insulins transfers the rate limitation of insulin absorption to other influencing factors, such as blood flow and diffusion distance to the vasculature, which are more readily influenced by acute exercise (55).

The decision to inject into a specific injection site around exercise may be hampered by logistical reasons (e.g. a rugby player removing their pump prior to a match, or an endurance cyclist unable to inject into the thigh during a ride) and also by a lack of knowledge as to any potential effects that are consequent of choosing one location over another. Few studies have compared the use of different injection sites during exercise. One study demonstrated the rate of absorption increased when injecting ^125^I-labeled actrapid into the exercising limb (thigh) compared to a non-exercising limb (arm) in people with T1D performing bouts of moderate-intensity bicycle exercise (20). However, this increase was only after exercise had ceased. Consistent with this finding, another study reported a greater average increase for the first 60 min of insulin absorption from the thigh (exercise 1.12 ± 0.12 vs rest 0.68 ± 0.15%.min^−1^; *p* < 0.05) than from the abdomen (exercise 0.87 ± 0.18 vs rest 0.75 ± 0.17%.min^−1^; *p* > 0.05) when healthy participants completed 20 min exercise on a cycle ergometer (19). The results of these studies, however, contrast those of another study using healthy participants in which significantly lower plasma insulin concentrations have been noted following 15 min of cycling after insulin actrapid injection into the thigh compared to the abdomen (25). However, the lack of data on statistical reporting, and the small (n = 4) sample size hinders the interpretation and applicability of this study. There is little data to confirm conclusions pertaining to injecting into the exercising limb and its effect on insulin pharmacokinetics. While injecting insulin into a site local to exercising muscle has been shown to accelerate absorption, injecting into a non-local site may also be subject to increased absorption (22).

The distribution of blood flow to the periphery for the purposes of thermoregulatory heat dissipation has been suggested as the underlying mechanism that explains the influence of temperature (40–43), and exercise (13, 22, 49, 56, 57), on the rise of the rate of insulin absorption. While this may apply to the rested individual, there is debate whether a temperature-induced increase in subcutaneous adipose tissue blood flow can solely account for the elevated rates induced by exercise. Upon starting exercise, blood flow is initially unchanged or shifted away from the skin towards the working muscles until thermoregulatory requirements stimulate the need for increased heat dissipation, due to elevated muscular metabolism, and blood flow to the skin begins to increase (58). As skin blood flow does not increase at the start (or, in hyperthermia, for its full duration (23)) of muscular activity, it is likely other factors contribute to the increased insulin absorption rate, which starts concurrently with the onset of exercise. Indeed, some authors detail an increase in insulin absorption despite no alterations in subcutaneous blood flow (19, 48). As the movement of blood through a section of subcutaneous-based capillary vessel interacts minimally with monomer movement in the interstitial fluid (due to the capillary membrane separating the interstitium and blood compartments), it is likely that blood flow *per se* does not directly impact the rate of insulin absorption. More probable, the rate-limiting step (after hexamer dissociation) is the ‘access’ insulin monomers have to capillary blood flow, gauged primarily by the diffusion distance from the depot to capillary endothelia. Vasodilation of terminal arterioles in subcutaneous tissue has been demonstrated to increase capillary recruitment, effectively increasing endothelial exchange surface area and potentially reducing monomer diffusion distance (59). However, this phenomenon is not yet fully elucidated in an exercise setting. While the zinc-hexamer association state has been shown to have higher thermic stability than the monomer state (60), to the authors’ best knowledge no studies have investigated the potential effects of temperature, separate to the concomitant effects of blood flow, on insulin absorption *in vivo*.

Diffusion of insulin into the circulation is dependent on the concentration gradient (i.e. a smaller concentration in the blood than the depot); hence, greater blood flow that transports insulin away from the vasculature, local to the depot, may indirectly promote the diffusion of insulin monomers away from the injection site by enabling a higher concentration gradient (55). Additionally, monomer movement may be influenced by a flushing effect of plasma volume movement into the local interstitium, or a massage effect from the underlying contracting muscle (61, 62). People with T1D should be aware that inter-individual differences may exist when injecting into exercising limbs, alongside the potential for increased rates of absorption.

**Sub-section conclusion:**

The exercise-induced increased rate of insulin absorption is likely due to a combination of factors relevant to the changes at injection site during exercise. The dissociation of insulin oligomers into biologically active units remains the initial rate-limiting step.

## Conclusion

Insulin absorption rate into circulation is influenced by different factors both at rest and during exercise. Compared to the same individual at rest, the exercise-induced increased appearance of insulin in the blood leads to a greater reduction in blood glucose. This phenomenon is often over-looked by individuals performing spontaneous bouts of activity or planning insulin adjustments around structured exercise. There is some evidence to suggest that the choice of location and depth of injection causes additional variability to absorption rates, whereby injections that are deeper and local to the working muscles are susceptible to even higher rates of insulin absorption. Overall, the cause of the increase in absorption during exercise is likely due to a myriad of factors including capillary recruitment, massage-effect, blood flow, temperature, and flushing effect; however, further studies are required to clarify their relative importance. Furthermore, the studies that have investigated the effects of exercise on absorption are now dated, using insulin types that are becoming increasingly less common among the T1D population. Studies using modern ultra-rapid and ultra-long acting insulins are required to determine whether the exercise-induced increase in the rate of absorption is still applicable. Patients and healthcare providers should be aware that the insulin pharmacokinetics around exercise may differ to resting profiles, enabling proactive avoidance of low blood glucose concentrations.

## Author Contributions

JP—Main investigator. Led literature search, draft composure, and draft review. OM—Aided literature search, draft composure, and draft review. TH-J—Aided draft composure, provided expert knowledge, and draft review. BW—Aided literature search, draft composure, and draft review. RB—Aided literature search, draft composure, and draft review and provided expert knowledge. All authors contributed to the article and approved the submitted version.

## Conflict of Interest

TH-J is an employee of Novo Nordisk A/S.

The remaining authors declare that the research was conducted in the absence of any commercial or financial relationships that could be construed as a potential conflict of interest.
